# Fused Deposition Modelling of Polymeric Auxetic Structures: A Review

**DOI:** 10.3390/polym15041008

**Published:** 2023-02-17

**Authors:** Davide Mocerino, Maria Rosaria Ricciardi, Vincenza Antonucci, Ilaria Papa

**Affiliations:** 1 Department of Chemical, Materials Engineering and Industrial Production, University of Naples Federico II, Piazzale Vincenzo Tecchio 80, 80125 Naples, Italy; 2 Institute for Polymer, Composites and Biomaterials, National Research Council, Piazzale Enrico Fermi 1, 80055 Portici, Italy

**Keywords:** additive manufacturing, 3D printing, auxetic structures, polymers, FDM

## Abstract

Additive Manufacturing (AM) techniques have recently attracted the attention of scientists for the development of prototypes with complex or particular geometry in a fast and cheap way. Among the different AM processes, the Fused Deposition Modelling process (FDM) offers several advantages in terms of costs, implementation features and design freedom. Recently, it has been adopted to realise auxetic structures, which are characterised by negative Poisson ratio, enhanced mechanical properties, and a higher compression resistance than conventional structures. This review outlines the use of AM processes, in particular FDM, to design and obtain auxetic structures, with the final aim to exploit their applications in different fields. The first part of this work presents a brief classification of auxetic structures and materials. Subsequently, a summary of additive manufacturing processes is presented, focusing on the use of FDM and its limitations. Finally, the studies on the use of additive manufacturing to produce auxetic structures are shown, evidencing the potential of the concurrent combination of a fast prototyping technique such as FDM and the characteristics of polymer- and/or composite-based auxetic structures. Indeed, this new technological field opens the possibility of realising novel structures with integrated smart behaviour, multifunctional properties, compression resistance, and a tailored microstructure and shape.

## 1. Introduction

**Figure 1:**
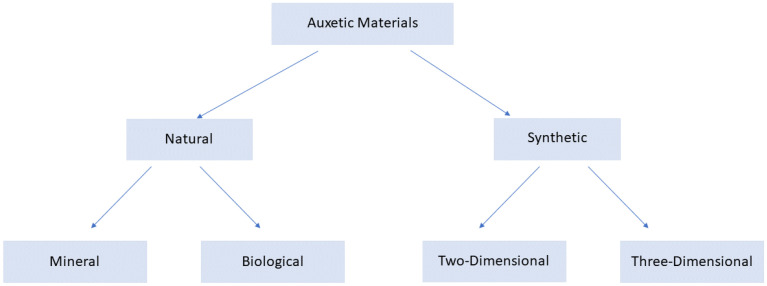
Classification of auxetic materials.

Auxetic materials are a class of fascinating materials that exhibit an unexpected behaviour when they are subjected to tensile or compressive loads [1]. Unlike conventional materials, in fact, they are characterised by a negative Poisson number, so when they are stretched axially, their thickness increases (Figure 1). Similarly, when they are subjected to uniaxial compression load, they contract transversely.

Starting from the concept of natural auxetic structures (α-Cristobalite, monocrystalline arsenic, tibia spongy bone, cat skin, salamander) and taking advantage of the natural improvement of the different properties [2,3,4], many studies focus on the design and fabrication of artificial auxetic structures for different fields of application [5].

With respect to the conventional materials, these structures exhibit several fascinating properties such as a better fracture resistance [6,7], an improvement of shear modulus [8,9], a variable permeability linked to the deformation [10,11], and an improvement of impact properties such as indentation resistance [12,13] and energy absorption capability [14,15] due to the lateral contraction of the impacted material, the consequent movement of the material in the impact area, and the corresponding increase in density. Therefore, auxetic structures can be used in many different applications such as defence [8], aerospace [16], biomedical [17], furniture, architecture and sports equipment [18]. Smith [19] and Baughman [4] found a way to take advantage of the negative Poisson ratio in metals using these materials as electrodes with a consequent amplification of the sensors’ response. Spadoni [20] exploited the behaviour of chiral structures to identify the deformation when subjected to dynamic load and to develop scalable geometry for aerodynamic applications. Bornengo investigated a similar concept to obtain a smart structure able to deform when subjected to airflow in order to apply them as a wing box of a racecar wing [21]. Other authors used the morphology of auxetic structures to develop tuneable filters able to change the permeability in a system by changing their pore-opening when subjected to determined loads [22,23].

Different techniques have been developed and improved to obtain auxetic structures with the aim to design and implement flexible, cost and time saving processes. The manufacturing of auxetic structures usually starts by modifying foaming and combining conventional processes such as compression, heating, and cooling. Several modifications have been exploited over time by using this fabrication process as a starting point, such as changing the mould’s geometry or using pins to fabricate larger samples [24]. Further, it is possible to produce composite auxetic materials by using particular stacking sequences [25,26], a specific reinforcement angle [27], auxetic constituents (matrix or reinforcement) [28]. For example, Subramani [29] used braided composite rods made of different fibres as a core of the auxetic structure and tested them using different angles of the geometry, showing the possibility of developing an auxetic structure at the macro-scale. However, conventional technologies can be limiting, even given the possibility of realising auxetic structures embedded with 3D auxetic reinforcements. In fact, they allow the production of composites with relatively poor mechanical properties (i.e., Young’s modulus) as compared with non-auxetic composites.

To overcome these limitations, additive manufacturing can be helpful, enabling the design of structures with complex geometry in a cheap and fast way and at a large scale. The development of additive manufacturing has currently reached a level of versatility that allows the design and optimisation of structures with a negative Poisson ratio and a multifunctional capability [30]. Schwerdtfeger [31] used a selective electron beam as an additive manufacturing technique to obtain Ti-6Al-4V auxetic structures in order to have a better control and variety of the geometry. Alomarah [32] studied different honeycomb auxetic structures by adopting a direct metal-printing technology and analysing the effect of the load direction. Selective laser melting was used by Lei et al. to prepare accurate auxetic structures made of AlSi10Mg [33]; he used this technology to study the connection between defects in production and the failure mode of the auxetic structure. Some additive manufacturing techniques, such as stereolithography, are used to realise precise and intricate large-size parts [34]. Others, such as direct laser writing, are usually considered to produce auxetic, small-size objects and micromechanical systems and microstructures, leading to a variety of dynamic devices for a large range of applications [35].

Among several AM techniques, the most suitable and versatile is the FDM process, which allows the production of polymer composite materials with negative Poisson ratios and many types of patterns [36].

One of the drawbacks of this technology is the large number of parameters that can influence the final results in terms of mechanical properties and mechanical behaviour, such as the failure mechanism. This was extensively reported by Vanaei in one of his works about the influence of parameters in FFF processes [37]. To better study these limitations, the same authors performed a large number of experiments based on response surface methodology (RSM) in order to predict the connection between the FFF parameters such as liquefier temperature, platform temperature, and print speed to the mechanical properties of the final manufacture. With this methodology, an optimised zone of extraction and an optimal range of parameters result in a better quality of the final part [38].

Furthermore, the growing evolution of this technology improves the design and development of a new class of auxetic composites with enhanced mechanical performance [39] in terms of stiffness and energy absorption. Consequently, different studies on auxetic materials’ mechanical behaviour obtained by the 3D printing process have been performed [40], opening new possibilities for the applications of auxetic reinforced composites. 

## 2. Auxetic Materials

The term auxetic comes from the Greek word “αűξεσις” or “auxesis” that means growth. The first research study on auxetic structures appeared at the beginning of 1990 [41], and was performed by Evans to describe materials with a negative Poisson ratio, following the foam work of Lakes [42] reporting a process to convert conventional foams with a positive Poisson ratio into auxetic foams.

In nature, examples of natural auxetic materials exist, and empirical evidence has been found for materials such as arsenic, antimony, and bismuth [43], α-cristobalite structures [44,45], and iron pyrites [46]. Grima discovered that this behaviour is frequently associated with a particular arrangement of the cellular structures [47], as in the case of the α-cristobalite and α-quartz [48], in which it is possible to observe an improvement of mechanical properties [16] due to the variation of the structural size between the nano- and macro-scale [49].

An easy classification of auxetic structures is reported in Figure 1.

**Figure 2:**
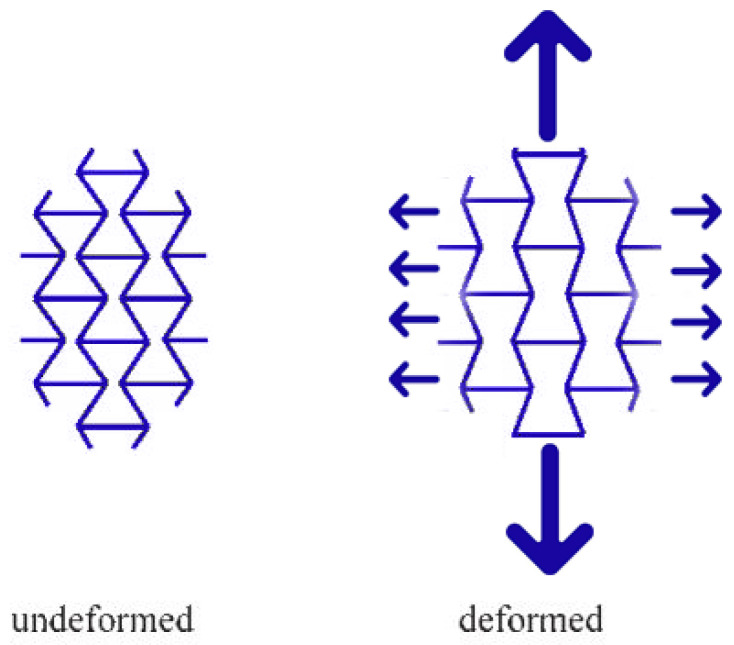
Different topologies of honeycomb auxetic structure reproduced from [50].

Honeycomb is the most common class of materials used to induce an auxetic behaviour inside a component [50]. In Figure 2 it is possible to observe the deformed configuration of a honeycomb structure due to a tensile loading.

**Figure 3:**
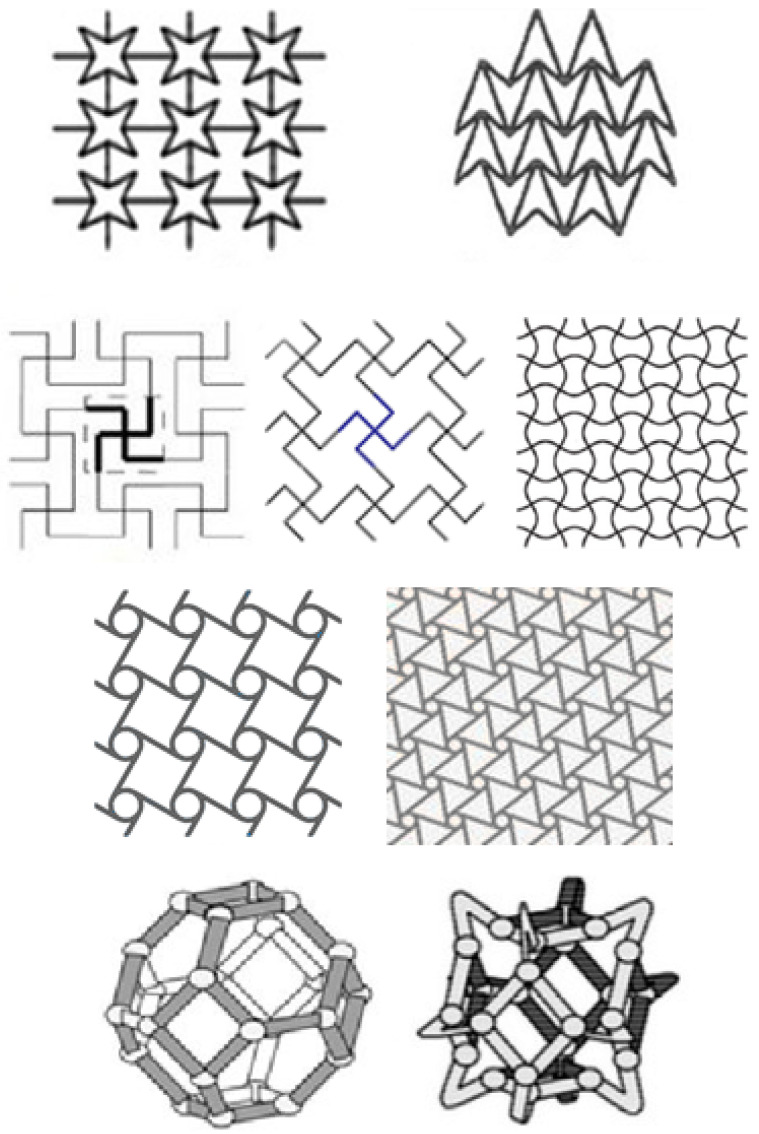
Different kind of auxetic topology reproduced from SAGE journals [65].

Different kinds of topologies can be realised depending on the geometry and on the required load [51] (see Figure 3).

Different studies have been performed on different structures by considering various auxetic models, which can be summarised as re-entrant structures, rotating deformation models, and chiral structures [52]. A re-entrant structure typically deforms by flexing diagonal ribs and, consequently, generating an outward unfold under tension and providing an auxetic behaviour to the structure. The rotating model consists of a complex system with rigid geometry [1]. Usually, it is reported in foams with a negative Poisson ratio. It comes from the rotation of the internal geometrical structure of the material subjected to a load, such as tensile deformation, causing an expansion in two directions [53]. Concerning the chiral structures, the lack of symmetric reflection is their principal characteristic; the auxetic effect is related to the wrapping or unwrapping of the connections around the nodes due to an applied force [54].

Guo et al. [55] analysed the importance of the orientation of re-entrant lattice cells in a lattice auxetic structure to obtain different deformations of the final product. Novak et al. [56] investigated the deformation behaviour of chiral auxetic structures at a high strain rate, finding that the plateau of the stress increased exponentially with the increase in load velocity. In addition, Novak et al. [56] analysed the mechanical behaviour of graded auxetic chiral structures, finding that the non-graded auxetic structure had a stiffer response due to the uniform deformation distribution among the specimens, but failed at lower strains compared to those with graded chiral structures. 

Starting from the basic structures, many researchers tried to improve the behaviour of auxetic structures by changing the structural topology. For example, Auricchio and Chen used a layered topological framework with an FEM approach [57] and embedded additional ribs into the existing structure to enhance its properties [58]. Chen et al. [58] performed a similar modification by introducing narrow ribs into a classic re-entrant structure, showing a significant increase in the elastic modulus and a higher yield stress, plateau stress and densification strain for the novel lattice structures. Foam is another kind of material that usually does not have a negative Poisson ratio but that, after proper processing, may exhibit an auxetic behaviour. Lakes [42] and Friis [59] studied, successfully, a way to obtain auxetic re-entrant foams from conventional foams, changing the structure by three different methods based on inwardly protruding cell ribs. Pickles studied the process parameters to develop auxetic materials using polyethylene by focusing on the compacting condition [60], on the sintering temperature and time [61], and on the extrusion condition [62]. Martz [63], indeed, tried to obtain auxetic foam, starting from closed-cell foams using air pressure for transformation. In 1997, Chan and Evans developed a way to produce a large auxetic foam block [64] by the processing of conventional polymeric foam in a multi-stage process. Successively, Wang and Lakes [65] found a relation between the cell size of the foam and the process parameters to obtain a negative Poisson ratio.

The auxetic structure appears to be helpful when the need is to improve some particular mechanical property. For example, chiral structures increase their resistance to global and local buckling with respect to the classic honeycomb [20,66], and some negative Poisson ratio foams are characterised by a higher energy absorption in dynamic impact than conventional ones; for example, they can improve their stiffness up to 4 times, their indentation stiffness up to 1.4 times and their energy absorption up to 3 times [13,67]. Further, they also show a better indentation resilience and a hardness that increases by 3 times with respect to conventional materials under the low-load condition [68,69]. Moreover, for some negative values of Poisson’s ratio, shear resistance will increase [70]. Fracture toughness can reach an increase of 225% [6,71], and fatigue life resistance [72] and stress–strain curves can be, in general, two orders of magnitude greater than those of the non-auxetic material [73]. The auxetic effect becomes even better after the yield point [74]. 

The electromagnetic and acoustic properties are enhanced too. Indeed, re-entrant honeycomb structures have more benefits than conventional honeycomb [75]. Howell [76] studied an auxetic foam as an acoustic absorber, discovering good properties. This was attributed to the microvibration of the foam cell ribs [77,78]. Finally, Alderson et al. [79] found that auxetic microporous polymers (ultra-high-molecular-weight polyethylene) have the capability to attenuate ultrasonic waves due to the dispersion of waves through the material. 

This section was used to describe in detail what an auxetic structure is. It is well explained how they were born, the traditional production methods and the necessity to obtain different mechanical properties using non-common geometry. A literature review is performed in order to categorise the different topologies, starting from 2D to the analysis of a 3D structure. A focus on the different uses of this kind of structure in different fields (from structural to acoustic absorption) is carried out in the last part of the section to better explain the necessity to design and produce these kinds of geometries.

## 3. Additive Manufacturing

**Figure 4:**
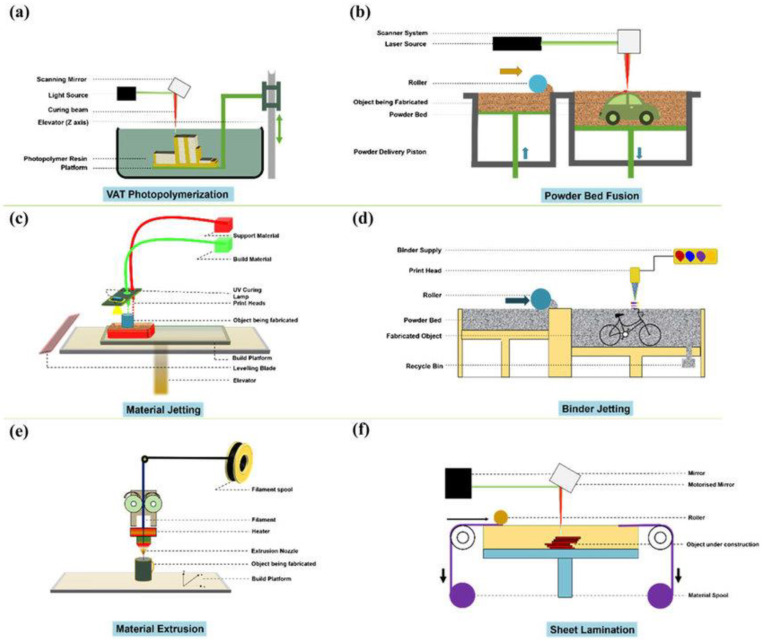
Different kinds of additive manufacturing: reproduced from SAGE journals [85].

Additive manufacturing allows the direct production of a model previously designed by using CAD 3D software; in this way, it is possible to realise the rapid prototyping of objects [80]. Furthermore, this technology enables the adoption of various materials and overcomes many limits of conventional processes. Different AM production methods have been recently developed (Figure 4): material jetting, material extrusion, sheet lamination, powder bed fusion, etc. [81,82,83,84].

Compared to the common production processes, the AM process offers a number of advantages such as the realisation of complex geometry, a lower “buy to fly” ratio, and design flexibility [86]. Due to these different benefits and the customising capability, related to the possibility of using a lot of materials, this process can be helpful for several fields and applications [87], such as the realisation of fibre-reinforced materials, combining different types of fibres and plastic matrices [88]. Furthermore, in the case of civil applications, 3D concrete printing allows one to obtain in situ walls and constructions with a robust design and manufacturing process [89], providing the possibility to combine reinforcement and metal components [90]. The intensive development of manufacturing technologies has resulted in new possibilities for making cellular structure models. Among the additive techniques used, PolyJet is a powerful 3D printing technology that produces finished and accurate objects, prototypes and tools. With microscopic layer resolution and accuracy down to 0.014 mm, it can produce thin walls and complex geometries using the widest range of materials available with any technology.

Among the advantages of this technology, we can mention the possibility of creating precise and detailed prototypes that perfectly render the aesthetics of the final product.

With this technology it is possible to produce moulds, jigs, and other production tools with great precision, and to create complex shapes with intricate details and refined elements. In [91,92], the cellular structure of polymer resins in the PolyJet Matrix additive technology were investigated. The influence of geometric structure shape on rheological properties was determined and the most favourable geometric variants of cellular structure models were determined. The rheological model was adopted and its parameters were determined.

PGJ incorporates the largest variety of colours and materials in a single model for unbeatable efficiency. In this regard, in [93], complex cellular structures made of three various photo-curable polymer resin types were manufactured. Materials were selected by taking into account the so-called “soft” and “tough” material groups. Compressive stress relaxation tests were conducted to match both printed model materials and their geometry in the future, to make a component with a specific rheological response.

The most popular materials processed by additive manufacturing are polymeric materials. Thermosetting, thermoplastic, hydrogel polymers and others are the usual materials involved in the production of systems, especially for biological environments [94] or for biomedical applications such as prosthesis and medical implants [95,96,97]. Nowadays, most studies are focused on developing biopolymers to improve the mechanical properties or the processability [98]. In addition, in recent years, the development of additive manufacturing has moved from simple 3D printing to 4D printing, to realise components with properties changing over time [99].

### Fused Deposition Modelling

**Figure 5:**
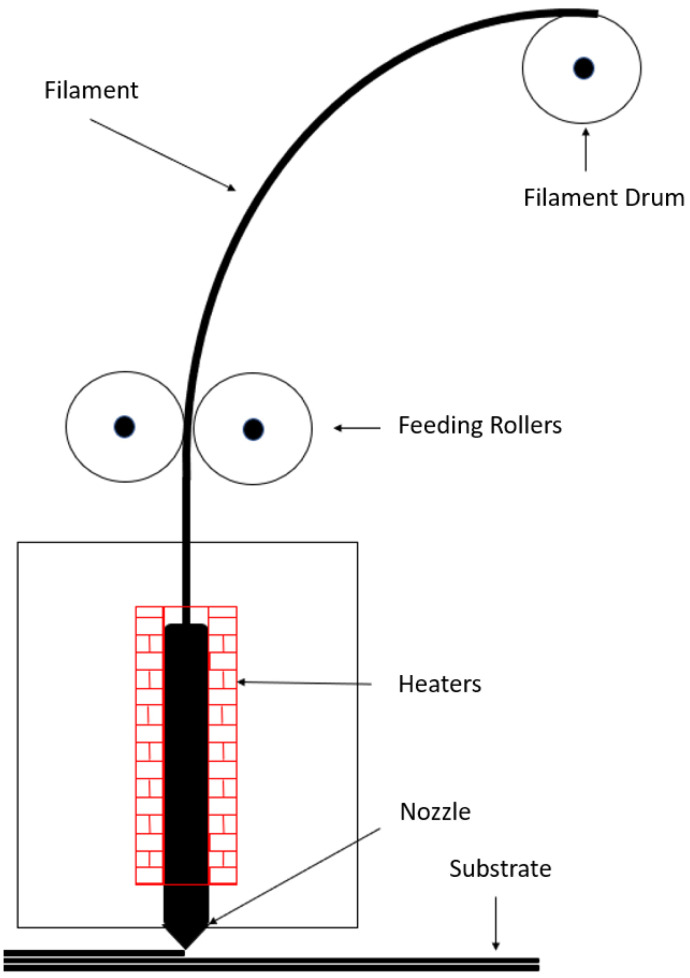
Schematic of fused deposition modelling machine.

At the end of 1980, starting from the experience of 3D printing, a new additive manufacturing technique, called the fused deposition model [100], was developed. The process (Figure 5) involves the extrusion of a heated plastic filament that becomes semi-molten and is deposited layer by layer directly from a digital model of the part onto a platform [101] to build the desired structure. 

This method is attracting the attention of scientists and engineers for several different applications due to its simplicity, cheapness, and low maintenance and investment costs, allowing the easy indoor development of the technology [102,103]. Common materials processed by this technique are thermoplastics, such as PLA (polylactic acid) [104,105] and ABS (acrylonitrile butadiene styrene) [106]. In addition, there are also some studies focusing on printing other kinds of polymers, such as PA (polyamide), PP (polypropylene), PC (polycarbonate) and PE (polyethene) [107]. However, the use of different kinds of thermoplastic materials with different melting temperatures requires the definition of the optimal printing parameters that need to be determined for each polymer. In this case, simulations can be helpful, allowing a faster and easier process design [108,109]. Obviously, the printing parameters and the polymer characteristics, such as the colour, affect [110] the mechanical properties, too. For example, the best tensile properties can be obtained by printing fibres parallel to the load direction [111,112]. 

One of FDM’s recent evolutions is the integration of polymeric materials and short or long fibres to obtain laminates and prototype composite materials in order to overcome the low mechanical properties of 3D-printed objects [113]. To this aim, new printing techniques of long fibres and new kinds of biopolymers have been developed [114,115]. For example, Matzuzaki et al. investigated the possibility to produce 3D-printed laminates with long fibres by performing the impregnation inside the printer’s nozzle [116,117]. Yang et al. [118] tried to mix normal PLA with poly(butylene succinate), obtaining a filament with stronger properties and more environmental features thanks to its energy-saving printing parameters. A similar study was carried out by Gkartzou [119] that tried to use lignin biopolymers based on PLA to obtain an eco-friendly polymer that improved commercial PLA’s properties. Mazzanti [120] reported different kinds of natural fibres used as a filler, their compatibility with different polymers, and the various parameters for printing. Duigou [121] used continuous flax fibres and a commercial printer to obtain a composite for structural applications, with the same longitudinal properties as those obtained with VARTM or thermocompression.

In general, as reported by Mazzanti et al. [122], most researchers have investigated the mechanical properties of FDM-printed composites based on PLA-, ABS- or polyolefin-based biocomposites, finding the average properties shown in Table 1.

The adoption of filaments with embedded short or continuous fibres can offer a slight or significant enhancement of the mechanical behaviour of 3D FDM-printed objects [113,117], respectively. As an example, Table 2 shows the results obtained by Matsuzaki et al. [117] for unreinforced PLA, as well as PLA reinforced with unidirectional jute fibres and with unidirectional carbon fibres.

The easy processability of FDM and the development of new biopolymers has increased the applications of filament fused deposition in many sectors such as automotive, aeronautical, naval, and above all biomedical [104,122,123]. The printing of cells, tissue, organ modules, and scaffolds has grown in recent years [124]. Some researchers, by modifying the normal process of FFD, developed particular printers to obtain healthy customised snack products [125]. Indeed, others focused on studying particular filaments to produce pharmaceutical products and pills [126,127]. 

This kind of technology has some drawbacks due to the processing parameters or printed materials. The principal one is the presence of voids in the final product; these defects can depend on the filament, which can have defects before printing, or on the process [102,128]. Furthermore, voids can induce a lack of adhesion between the printed layers, and structural defects that influence the mechanical properties and the quality of final part [129]. Other imperfections of the printed objects are: stepped layers, stringing, warping, structural inhomogeneity, and hygroscopic [103,130]. To avoid problems such as porosity and voids, Gordeev et al. [131] found better printing parameters by acting on G-code and wall thickness, thereby increasing the quality and properties of the final objects. 

In this section, a review of additive manufacturing technology is reported. It is a useful tool for the direct production of 3D models using CAD software, enabling rapid prototyping with various materials. The different kind of AM methods are described, such as material jetting, material extrusion, powder bed fusion, etc. Additionally, the advantages of using AM in various application fields are well reported based on past studies. A report of different common materials used with this technology is performed and future prospects in terms of 4D printing are shown. Special attention is paid to filament deposition moulding, focusing on the different applications and on its evolution in order to integrate polymeric composite materials to improve the mechanical properties of 3D-printed objects. A report of the mechanical properties of common printed polymers is shown and the drawbacks of this technology are presented.

## 4. Additive Manufacturing for Polymeric Auxetic Structures

The additive manufacturing technique allows the possibility of designing and realising novel 2D and 3D auxetic structures [132,133,134,135,136] based on polymeric materials, increasing their potential and applications. In fact, starting from different production methods, several scientists focused on developing polymeric materials to obtain auxetic structures with improved elastic deformation. Agnelli et al. [137] developed a procedure to optimise the Poisson ratio of an auxetic structure and to realise it by a rubber-like polymer GM08b using a stereolithography process. The obtained structure showed a linear response of up to 5% of strain despite the non-linear behaviour of the base polymer in the same range. A photo-stereolithography technique was also used to produce a tuneable robust scaffold by combining a polyaliphatic urethane acrylate blend with isobornyl acrylate to obtain a final polymer with regional auxetic behaviour and with an enhanced elastic deformation [138]. Pandini et al. [139] used a photopolymer resin by the addition of a photo-initiator [140]) to obtain shaped memory 4D-printed auxetic structures with a 95% strain recovery after deformation, providing a great improvement in the production of biomedical components and robotics. Wong et al. [141] developed an ionogel ink that can be printed into geometric objects that are ionically conductive and used it to realise a strain sensor with auxetic geometry, high ionical conductivity, an extension of 310% more than a conventional continuous film, and a tolerance to internal failure [141]. A similar process was used by Wagner et al. [142] that used a photopolymer printing process and a polymer with shape memory properties (VeroWhitePlus RGD835) to obtain a metamaterial able to sustain changes in area of up to 200%. The evolution of photopolymeric printing was used by Wu [143], who used a grayscale pattern to obtain a 4D auxetic structure made from a polymer realised by the mixture of three different materials (polydiacrylate–butymethacryate and butylacrylate). Thanks to this process, it was possible to obtain active structures with a reversible pattern transformation. Among the AM technologies, the multi-jet fusion process allowed the creation of polyamide 12 auxetic structures with different topological cell units and deformation behaviours. The realised structures showed high values of energy absorption and the closest behaviour to the ideal energy absorber [144] under compression tests. Furthermore, it is possible to use laser sintering systems to manipulate thermoplastic polyurethane auxetic structures and obtain significant recoveries in terms of strain; for example, it is easy to go from 100% of deformation to 25% in a relaxed state, and from 200% and 300% to about 100% in a relaxed state [145].

### FDM Auxetic Structures

The use of FDM for the production of novel auxetic structures is a frontier theme that is attracting the attention of several researchers due to the possibility of studying, designing and realising structures with enhanced compression properties, crack-absorbing capabilities, and tuneable mechanical properties. In particular, thanks to the possibility of coupling this new approach with conventional technologies, it is possible to properly modify some properties in the final product [146]. For example, Grimmelsmann et al. [146] combined 3D printing and knitted technology to realise novel metamaterials with improved permeability and mechanical properties. They used 3D nylon auxetic structures to modify the pore size and shapes of textile fabrics and improved the stress–strain behaviour up to 60 times, obtaining a stable connection between the two materials. Further, the work of Quan et al. [147] evidenced the low cost and rapid manufacturing of a thermoplastic polymer reinforced with carbon-fibre auxetic structures by using an FDM process; the realised products showed an increase in compressive stiffness, energy absorption and, due to the fibres’ presence, the capability to stop crack propagation and prevent breakage compared to pure PLA auxetic structures.

Vyavahare, with the help of numerical simulations [148,149], investigated the behaviour of 3D-printed PLA and ABS re-entrant auxetic structures and observed mechanical resistance changes as a function of printing parameters. Alomarah et al. [150] studied, for the first time, the properties of the printed re-entrant chiral auxetic structure by comparing them with traditional auxetic structures and found an improvement of Young’s modulus and out-of-plane compression properties. Khare [151] used PLA to realise high-strain, tuneable 2D and 3D auxetic materials with distributing stress and strain properties, and the capability to switch easily between negative and positive Poisson ratios. The performance of the novel structures having an s-hinged unit cell was validated by a semi-analytical approach that allowed the prediction of the structures’ mechanical properties. PLA was also used to realise, by printing, lattice structures with tuneable mechanical properties after fabrication [152]. Kim et al. [153] focused on the effect of the selected thermoplastic material and its hardness and mechanical properties on the negative Poisson ratio. Lvov [154] developed an auxetic honeycomb structure able to increase the low cycle compression resistance up to two times with respect to the similar non-auxetic structure, showing a better distribution of load after cyclic tests and a better capability for energy dissipation. FDM technology is also useful for obtaining hybrid chiral mechanical metamaterials using multi-material 3D printers that allow the possibility of producing smart composites that are able to adapt to external loads and conditions [155,156]. One of the most interesting applications of biocompatible auxetic structures realised by the FDM method is the production of nasopharyngeal swabs for COVID-19 detection. In particular, Arjunan et al. [157] improved the design of nasopharyngeal swabs, reducing the pain and the discomfort by taking advantage of the properties of auxetic materials to shrink under axial resistance. The advantage of using FDM in prototyping is the simplicity of obtaining auxetic structures and of changing some parameters in order to study the effects of the geometrical parameters on the properties of whole structures [158,159,160]. FDM for auxetic structures is absolutely suitable to develop new 3D-printed smart structures able to change shape. In this case, the procedure is known as 4D printing [161]. Dong [162], for example, fabricated novel electro-induced shape memory auxetic composites to realise innovative components such as orthopaedic devices that are able to adapt to electrical stimulation. 4D printing has been used to obtain meta-sandwich polymers with large values of energy absorption (up to 38% of the total energy) by combining the Flex-Pro soft polymer with the harder polyurethane-based one [163]. 

The previous section explains in detail the use of additive manufacturing technology to design and create novel 2D and 3D auxetic structures using polymeric materials, improving their potential and applications. Scientists have used different production methods to develop polymeric materials to obtain auxetic structures with improved elastic deformation. This includes using stereolithography, photo-stereolithography, photopolymer resin, ionogel ink, laser sintering systems, and the multi-jet fusion process. These methods have resulted in structures with improved elasticity, energy absorption, and reversible pattern transformation. Especially, FDM has gained interest for the production of novel auxetic structures. A report of different auxetic structures produced by FDM coupled with other conventional technologies is shown, and the contributions of many researchers in the use of thermoplastic polymers for 3D-printed auxetic structures with improved mechanical properties are mentioned. Different applications are shown, such as the production of biocompatible auxetic structures and further works such as 4D-printed smart structures.

## 5. Conclusions

This review discusses the AM processes for the design and production of auxetic structures. First, a brief series of auxetic structures and materials is reported in order to give an overview of the most proper structures for a specific field. Then, the additive manufacturing processes are described, considering their historical background, advantages and disadvantages. Finally, the fused deposition modelling (FDM) technique and its limitations are discussed to outline the current state of the art on new applications for auxetic structures realised by FDM. 

FDM is a special technique based on material extrusion that provides several AM benefits. It allows the customisation of tailored items, promotes design elasticity, and reduces the difficulty of complex element production for complex parts, reducing costs and lead times for goods development. On the other hand, FDM offers other significant benefits in terms of cheap and low-maintenance machines and low-cost stock materials compared to other AM techniques. Further, it offers the possibility of modifying the components’ properties and developing materials with nature-inspired structures.

Actually, studies on the use of AM and FDM to manufacture auxetic polymeric structures have been mainly performed to exploit the potential of auxetic structures. These particular structures have interesting mechanical properties that appear helpful for biomedical or for energy absorption applications. Further, the novel emerging AM techniques suggest the opportunity to produce unconventional structures and have great potential to realise continuous fibre-reinforced composite lightweight structures with complex shapes, interesting mechanical properties, and multifunctional and smart attributes. 

To this aim, since some critical processing issues are still present, a great effort is required to overcome the technological limits and to develop scalable techniques able to produce auxetic structures with tailored microstructures and properties and to move from small-scale to industrial products.

However, the innovation in the world of 3D printing occurs quickly. New applications are tried out, new materials are tested and adapted to the technology, and new hardware is developed and marketed. However, despite this continuous innovation, a new technology seems to be appearing that incorporates and almost surpasses additive manufacturing: 4D printing, in which the fourth dimension is time. “4D printing” means the moulding of intelligent and programmable materials, i.e., capable of changing shape, properties or performing specific actions such as, for example, self-assembling over time, following an external stimulus such as heat, vibration, gravity, magnetism or electricity. It is a further step forward that expands the already wide possibilities offered by additive manufacturing, which was developed with the aim of simplifying the infrastructures and mechanisms commonly employed in the design of programmable or self-assembling elements. The technology also makes it possible to break down one of the major constraints of 3DP: the print volume. Today, in fact, it is only possible to obtain smaller objects from the printer, while 4DP instead allows the creation of artefacts in a compact configuration, that are able to expand and acquire a different shape or larger dimensions as a result of a pre-set stimulus during the design. It all started from the self-assembly lab, a laboratory at the famous Massachusetts Institute of Technology (MIT), coordinated by architect/designer/computer scientist Skylar Tibbits who, in April 2013, presented a preview of this innovation at the TED conference (Technology Entertainment Design). The basic principle is scalable; therefore, the number of possible applications is enormous, from the nano- and micrometric scale to biomedical implants that compress when inserted into the body—and once inside they recover their shape, perhaps to repair an artery—up to the design of bridges and infrastructures.

The Tibbits team has already partnered with Stratasys, through which a printable material has been developed capable of activating in contact with water, and consequently changing shape, with which an element has been created that, once immersed, reacts and assumes the configuration of a cube. The closest development of this principle will be the possibility of exploiting these characteristics to decrease the size of objects during storage and shipment, in order to be more practical and economical. Once it has arrived at its destination, the product can be activated and resumes its original shape, or takes on a new one, acquired according to the context in which the object is found, in order to optimise its performance. An example could be a solar panel, folded during shipment, which, once installed, opens and automatically positions itself to optimise the reception of the sun’s rays. As is often the case, with a new technology, it is the military sector that offers resources for research. In this case, the US Army has allocated $855,000 for the development of textile materials capable of changing their structure in order to produce intelligent uniforms, capable of adaptive and automatic camouflage. 4D printing offers some really interesting opportunities, and its possible evolutions can be amazing.

## Figures and Tables

**Table 1:** Average tensile properties of 3D-printed samples based on different polymeric matrices.

Polymer	Strength, MPa	Stiffness, GPa	Elongation at Break, %
PLA	30 ± 10	2 ± 1	6 ± 4
ABS	25 ± 5	1.5 ± 0.5	5 ± 1
PE/PP	20 ± 10	1 ± 2	2.5 ± 0.5

**Table 2:** Average tensile properties of 3D-printed samples based on unreinforced and reinforced PLA.

Material	Tensile Strength, MPa	Tensile Modulus, GPa	Elongation at Break, %
Unreinforced PLA	30.9 ± 5	4 ± 1	1.45 ± 0.0945
Juta fibres/PLa	57.1 ± 5.3	5.11 ± 0.41	1.7 ± 0.6
Carbon fibres/PLA	185.2 ± 24.6	19.5 ± 2.08	0.95 ± 0.0873

## Data Availability

The raw/processed data required to reproduce these findings cannot be shared at this time due to technical or time limitations.
